# Genetic Polymorphisms in *SORL1*, *CLU*, *BIN1*, and *VDR* Are Associated with Mild Cognitive Impairment and Dementia in a Russian Population

**DOI:** 10.3390/diseases14100368

**Published:** 2026-10-03

**Authors:** Danil Peregud, Olga Pavlova, Konstantin Pavlov, Kira Nebogina, Yana Zorkina, Olga Abramova, Valeriya Ushakova, Aleksandra Ochneva, Eugene Zubkov, Inna Shamakina, Alisa Andryushchenko, Georgy Kostyuk, Anna Morozova, Olga Gurina, Vladimir Chekhonin

**Affiliations:** 1 V. Serbsky National Medical Research Centre of Psychiatry and Narcology, 119034 Moscow, Russia; peregud_d@yahoo.com (D.P.); pavlova.o@serbsky.ru (O.P.); pkanew@mail.ru (K.P.); kira.nebogina@mail.ru (K.N.); zorkina.ya@serbsky.ru (Y.Z.); ushakovavm@yandex.ru (V.U.); aleksochneva@yandex.ru (A.O.); zubkov.e@serbsky.ru (E.Z.); shamakina@yahoo.com (I.S.); hakurate77@gmail.com (A.M.);; 2 Mental-Health Clinic No. 1 Named After N.A. Alekseev, 115191 Moscow, Russia; 3 Faculty of Psychology, Lomonosov Moscow State University, 119991 Moscow, Russia; 4 I. M. Sechenov First Moscow State Medical University ( Sechenov University), 119435 Moscow, Russia; 5 Department of Medical Nanobiotechnology, Institute for Translational Medicine, Pirogov Russian National Research Medical University, 117997 Moscow, Russia; 6 Endocrinology Research Centre, 117292 Moscow, Russia

**Keywords:** mild cognitive impairment, dementia, Alzheimer’s disease, genetic polymorphism, BIN1, SORL1, CLU, VDR, Russian population

## Abstract

Background: Mild cognitive impairment (MCI) is a transitional state between normal aging and dementia, and genetic factors may influence progression. This study aimed to identify genetic polymorphisms associated with MCI and dementia in a Russian population. Methods: A total of 280 participants were enrolled: Controls (*n* = 84), MCI (*n* = 94), and Dementia (*n* = 102). Fifteen single nucleotide polymorphisms in seven candidate genes (TOMM40, SORL1, BIN1, VDR, CLU, CR1, PICALM) were genotyped. Associations were tested using multinomial logistic regression adjusted for age and sex under four genetic models (codominant, dominant, recessive, overdominant). Multiple testing was controlled using the Benjamini–Hochberg false discovery rate (FDR) procedure. Deviation from Hardy–Weinberg equilibrium was observed for three SNPs in the Controls (SORL1 rs1010159, BIN1 rs7561528, rs10200967) and interpreted with caution. Results: Under the primary FDR correction (60 tests per contrast), only BIN1 polymorphisms remained significantly associated with dementia: rs7561528 (overdominant model: OR = 0.318, 95% CI = 0.162–0.624, *p* = 0.001, *q* = 0.0035) and rs10200967 (overdominant model: OR = 0.272, 95% CI = 0.136–0.544, *p* < 0.001, *q* < 0.007). Under the more conservative exploratory correction (150 tests), these associations were at the threshold of significance (*q* = 0.050). Conclusions: BIN1 rs7561528 and rs10200967 are associated with reduced odds of dementia in the Russian population. Nominally significant associations with SORL1, CLU, and VDR require replication in larger cohorts. The developed genotyping panel represents a cost-effective research tool for genetic screening; clinical implementation requires validation in larger prospective cohorts.

## 1. Introduction

Dementia is a major public health challenge worldwide, with Alzheimer’s disease (AD) being the most common cause, accounting for 60–70% of all dementia cases [1]. The disease is characterized by a progressive neurodegenerative process with predominant involvement of brain structures responsible for memory, thinking, speech, and behavioral regulation, leading to loss of cognitive and social functions and reduced quality of life [2]. In the Russian Federation, the number of patients with AD exceeds 1.9 million, with the actual prevalence likely substantially higher due to underdiagnosis of early-stage cognitive impairment [3].

Particular attention in contemporary research is devoted to the stage of mild cognitive impairment (MCI), considered a prodromal phase of dementia [4]. Although not all patients with MCI progress to dementia, this stage offers the greatest potential for preventive and therapeutic interventions [5]. Therefore, the identification of objective biomarkers enabling prediction of cognitive decline progression represents a critical scientific task.

Current evidence indicates that cognitive impairment is multifactorial. It arises from the interaction of genetic and environmental factors [6]. Genetic polymorphisms may directly or indirectly modify the functioning of key neuronal systems, contributing to individual differences in the risk of cognitive decline and its rate of progression [7]. Large-scale genome-wide association studies (GWASs) have identified numerous loci associated with AD and dementia. To date, 101 independent variants in 81 loci have been identified in European populations [8]. Meta-analyses conducted by the Alzheimer’s Disease Genetics Consortium (ADGC) have confirmed that loci including clusterin (CLU), complement receptor type 1 (CR1), and phosphatidylinositol-binding clathrin assembly protein (PICALM) genes are associated with AD risk at genome-wide significance [9,10,11].

The *CLU* gene encodes CLU, a chaperone protein involved in amyloid-β clearance, complement regulation, and neuroinflammation [12,13]. Elevated plasma CLU levels have been observed in MCI and AD patients, and the rs11136000 variant has been consistently associated with AD risk across multiple populations [14,15]. The *CR1* gene encodes CR1, which mediates complement activation and immune complex clearance. Variants in *CR1*, including rs6656401 and rs1408077, have been linked to AD risk, likely through modulation of complement-mediated synaptic pruning and neuroinflammation [16,17]. *PICALM* encodes a clathrin assembly protein involved in endocytosis and intracellular trafficking. The rs541458 variant has been associated with AD risk, potentially affecting amyloid precursor protein (APP) processing and synaptic function [18,19].

Beyond these well-established GWAS loci, polymorphisms in translocase of outer mitochondrial membrane 40 (*TOMM40*), sortilin-related receptor 1 (*SORL1*), and bridging integrator 1 (*BIN1*) have emerged as promising candidates. *TOMM40* encodes the TOMM40 protein, which is involved in mitochondrial protein import. The rs2075650 variant, located in an intronic region near Apolipoprotein E (APOE), has been associated with AD risk and cognitive aging, likely reflecting its role in mitochondrial dysfunction and energy metabolism [20,21]. *SORL1* is involved in APP trafficking, and its variants have been associated with AD risk and progression from MCI to dementia [22,23,24]. *BIN1* is a GWAS-confirmed risk locus for AD, associated with tau pathology and cognitive dysfunction [25,26,27]. The variants rs744373, rs7561528, and rs10200967 have been linked to AD risk and represent the second most significant GWAS loci [28,29].

The vitamin D receptor (VDR) gene, widely expressed in the substantia nigra, cortex, thalamus, and hypothalamus, has been associated with MCI, AD, and Parkinson’s disease [30]. Vitamin D regulates signaling in septohippocampal pathways through neurotrophic factors and modulates expression of genes involved in acetylcholine, dopamine, serotonin, and gamma-aminobutyric acid (GABA) synthesis [31,32]. *VDR* polymorphisms *BsmI* (rs1544410) and *ApaI* (rs7975232) are functional regulatory variants affecting receptor expression [33]. 

It should be noted that the GWAS-derived variants in *CLU* (rs11136000, rs9331888), *CR1* (rs6656401, rs1408077), *PICALM* (rs541458), *TOMM40* (rs2075650), *SORL1* (rs2070045, rs1010159, rs3824968, rs2282649), and *BIN1* (rs744373, rs7561528, rs10200967) are predominantly intronic tag SNPs marking associated chromosomal regions; the functional variants responsible for these associations remain to be identified. This reflects a broader challenge in GWAS interpretation, where linkage disequilibrium obscures causal variants [34], and fine-mapping approaches are required to prioritize credible causal variants [35].

Despite these advances, the vast majority of genetic association studies have been conducted in non-Russian populations [36,37,38]. Data on the distribution and clinical significance of these polymorphisms in the Russian population remain scarce [3]. Moreover, existing diagnostic and therapeutic developments in the field of dementia primarily focus on pharmacological symptomatic therapy and neuroimaging, while the number of diagnostic tools incorporating genetic markers for risk prediction remains limited [38,39]. This gap underscores the need for population-specific genetic research.

Based on these considerations, the present study was designed with the following objectives: to evaluate associations between 15 candidate genetic polymorphisms in *TOMM40*, *SORL1*, *BIN1*, *VDR*, *CLU*, *CR1*, and *PICALM* and the risk of MCI and dementia in a Russian population.

We hypothesized that polymorphisms in genes regulating key biological processes—including lipid metabolism, endocytosis, neuroinflammation, and neurotrophic signaling—play a significant role in the development of cognitive impairment. The candidate gene approach, utilizing real-time polymerase chain reaction (PCR)-based genotyping, was selected as a technically accessible, cost-effective, and well-validated method.

## 2. Materials and Methods

### 2.1. Study Design and Participants

This observational retrospective case–control study was conducted at the Federal State Budgetary Institution “National Medical Research Centre for Psychiatry and Narcology named after V.P. Serbsky” of the Ministry of Health of the Russian Federation. The study protocol was approved by the local ethics committee, and all participants provided written informed consent prior to enrollment.

A total of 280 participants of self-reported European ancestry, aged under 75 years, were enrolled and allocated into three groups:

1. Control group (*n* = 84): participants without cognitive impairment (Montreal Cognitive Assessment (MoCA) score ≥ 26), with no signs of alcohol abuse.
2. MCI group (*n* = 94): patients diagnosed with mild cognitive impairment (International Statistical Classification of Diseases and Related Health Problems, 10th Revision (ICD-10): F06.7).
3. Dementia group (*n* = 102): patients with clinically confirmed dementia, including Alzheimer’s disease (ICD-10: F00*/G30.- †), vascular dementia (ICD-10: F01), and dementia in other diseases (ICD-10: F02*). However, the majority of them had a diagnosis of F02.8 (mixed dementia), and only a few had other specified dementia subtypes.

Inclusion criteria were 18–75 years of age and having signed informed consent.

Exclusion criteria for all groups were comorbid conditions associated with epilepsy, oncological diseases, or endogenous psychiatric disorders; dementia of infectious origin (HIV, neurosyphilis); dementia of traumatic origin; refusal to participate at any stage; onset of exclusion criteria preventing continued participation. For the Control group, additional inclusion criteria were the absence of cognitive impairment according to MoCA, as well as the absence of alcohol abuse.

### 2.2. Clinical and Psychometric Assessment

Cognitive function was assessed using MoCA. MoCA is a screening tool for cognitive impairment, evaluating multiple domains including attention, concentration, executive functions, memory, language, visuoconstructional skills, conceptual thinking, calculations, and orientation.

MoCA was used to distinguish between the Controls (score ≥ 26) and participants with MCI (score 18–25 with subjective cognitive complaints), in accordance with standard MoCA interpretation. Both groups were recruited from outpatient settings. The Dementia group was recruited separately from a psychiatric hospital and comprised severely affected inpatients who were unable to complete the MoCA. Clinical diagnoses of MCI and dementia were established by qualified psychiatrists and neurologists according to ICD-10 criteria.

### 2.3. Biological Sample Collection and Processing

Peripheral venous blood samples (5 mL) were collected from all participants on the day of enrollment under fasting conditions. Blood was drawn into EDTA-containing vacutainer tubes and processed within 4 h of collection.

Samples were centrifuged at 1000× *g* for 15 min at 4 °C. The plasma and buffy coat fractions were separated and stored at −80 °C until further analysis. Genomic DNA was extracted from whole blood samples using the “M-Sorb” DNA extraction kit (Syntol, Moscow, Russia) on an automated KingFisher Flex System (Thermo Scientific, Waltham, MA, USA) according to the manufacturer’s protocol.

The extraction procedure involved the following steps:

(1) Cell lysis using a chaotropic agent to release DNA
(2) DNA binding to silanized magnetic particles in the presence of a precipitating reagent
(3) Washing steps to remove contaminants
(4) DNA elution using a low-salt buffer
(5) Separation of DNA solution from magnetic particles by magnetic force

DNA concentration and purity were assessed spectrophotometrically using a NanoDrop 2000 (Thermo Scientific, Waltham, MA, USA). Samples were normalized to a final concentration of 10 ng/μL for subsequent genotyping. DNA samples with A260/A280 ratios between 1.8 and 2.0 were considered suitable for analysis.

### 2.4. SNP Selection and Genotyping

A total of 15 single nucleotide polymorphisms (SNPs) in seven candidate genes were selected based on prior GWAS findings and the literature evidence of association with AD, MCI, or cognitive impairment. The selected genes and their biological functions are summarized in Table 1.

DNA extraction and genotyping were performed as described previously [40] using the “SNP-Screen” reagent kits based on TaqMan chemistry (Syntol, Moscow, Russia) on a QuantStudio™ 5 Real-Time PCR System (Thermo Fisher Scientific, Waltham, MA, USA) according to the manufacturer’s instructions. All genotyping assays were performed in duplicate, and a subset of 10% of samples was randomly selected for repeat analysis to verify reproducibility. Allele discrimination was performed using the QuantStudio™ Design and Analysis Desktop Software Version 1.5. Only samples with high-quality PCR amplification and clear allele discrimination were included in the final statistical analysis.

### 2.5. Statistical Analysis

Statistical analysis was performed using IBM SPSS Statistics version 23.0 (IBM Corp., Armonk, NY, USA) and Prism 6 for Windows (GraphPad Software Inc., Boston, MA, USA). Continuous variables were tested for normality using the Kolmogorov–Smirnov test. Data were expressed as median [interquartile range] for non-normally distributed variables.

Hardy–Weinberg equilibrium (HWE) was assessed for each SNP in each group using the χ^2^ goodness-of-fit test with one degree of freedom. Deviation from HWE was considered significant at *p* < 0.05. The minor allele was defined as the less frequent allele in the study population.

Group comparisons of continuous variables were performed using Kruskal–Wallis one-way ANOVA followed by Dunn’s post hoc test for multiple comparisons (three or more groups).

Genotype and allele frequency comparisons between groups were performed using the χ^2^ test (or Fisher’s exact test with expected frequencies <5). For each SNP, four genetic models were tested:

- Codominant model: each genotype compared individually.
- Dominant model: minor allele carriers (heterozygotes + minor homozygotes) vs. major homozygotes.
- Recessive model: minor homozygotes vs. major homozygotes + heterozygotes.
- Overdominant model: heterozygotes vs. both homozygotes combined.

Association analysis was performed using multinomial logistic regression with the Control group as reference. Age and sex were included as covariates in all regression models. Results were expressed as odds ratios (ORs) with 95% confidence intervals (95% CIs). A two-tailed *p*-value < 0.05 was considered statistically significant for nominal associations.

Correction for multiple testing: Given the exploratory nature of this study and the relatively small sample size, we applied the Benjamini–Hochberg false discovery rate (FDR) procedure [6] to control for multiple comparisons across the 15 SNPs tested. An FDR-adjusted *q*-value < 0.05 was considered statistically significant. Both nominal *p*-values and FDR-adjusted *q*-values are reported. We note that the 150-test correction is highly conservative and substantially increases the risk of type II error, as it includes analyses that were not part of the primary hypothesis.

### 2.6. Ethical Considerations

The study was conducted in accordance with the ethical principles of the Declaration of Helsinki (1975, revised in 2008) and the national ethical standards for human research. The protocol was reviewed and approved by the Medical Ethics Committee of the National Medical Research Centre for Psychiatry and Narcology named after V.P. Serbsky. All participants or their legal representatives provided written informed consent prior to enrollment. Participants were informed of their right to withdraw from the study at any time without consequences for their medical care.

## 3. Results

### 3.1. Demographic and Clinical Characteristics of the Study Population

A total of 280 participants were included in the study: 84 in the Control group, 94 in the MCI group, and 102 in the Dementia group. Demographic and clinical characteristics are presented in Table 2.

Significant differences were observed between groups in age (Kruskal–Wallis H = 9.77, *p* < 0.0076), sex distribution (χ^2^ = 70.2, *df* = 2, *p* < 0.0001), and MoCA scores (Kruskal–Wallis H = 176.7, *p* < 0.0001). Participants in the MCI group were older as compared with the Dementia group (*p* = 0.007). The Dementia group had a higher proportion of males (52.9%) compared to the MCI (8.5%) and Control (4.8%) groups. MoCA scores decreased progressively from Control (median 27) to MCI (median 22) to Dementia (median 9), with all pairwise comparisons reaching statistical significance (*p* < 0.001 for all).

### 3.2. Hardy–Weinberg Equilibrium and Genotype Frequency Distribution

HWE was assessed for each SNP in each group (Table 3). Deviation from HWE was observed in the Control group for three SNPs: SORL1 rs1010159 (χ^2^ = 7.66, *p* = 0.022), BIN1 rs7561528 (χ^2^ = 9.18, *p* = 0.010), and BIN1 rs10200967 (χ^2^ = 12.20, *p* = 0.002). All other SNPs were in HWE in all groups (*p* > 0.05).

Genotype and allele frequencies are presented in Table 3. Significant differences in genotype distribution were found for BIN1 rs7561528 and rs10200967 between Dementia and Control (*p* = 0.003 and *p* = 0.001, respectively). No significant allele frequency differences were observed after correction for multiple testing.

### 3.3. Association of Genetic Polymorphisms with MCI and Dementia

Associations between each SNP and disease status were assessed using multinomial logistic regression adjusted for age and sex, with the Control group as reference. Four genetic models were tested: codominant, dominant, recessive, and overdominant. Nominally significant associations are summarized in Table 4. Full results for all SNPs and models are provided in Supplementary Table S1.

After FDR correction for multiple testing (150 tests), none of the nominally significant associations (*p* < 0.05) survived and should be considered exploratory. However, *BIN1* rs7561528 and rs10200967 demonstrated a clear trend for significance (*q* (FDR) = 0.050).

### 3.4. Sex-Specific Association of Genetic Polymorphisms with MCI and Dementia

Due to substantial imbalance in sex distribution across groups, we performed additional analyses restricted to women. Similar to the total cohorts, significant differences in genotype distribution were found for BIN1 rs7561528 and rs10200967 between the Dementia and Control groups (*p* = 0.005 and *p* = 0.002, respectively; Supplementary Table S2). As in the case of total cohorts, logistic regression demonstrated that *BIN1* rs7561528 and rs10200967 were nominally associated with dementia as compared with Control (Table 5). However, after FDR correction for multiple testing (150 tests), none of the nominally significant associations (*p* < 0.05) survived. Results of logistic regression for all SNPs and models are provided in Supplementary Table S3. Exactly the same pattern of *BIN1* rs7561528 and rs10200967 associations with dementia was demonstrated in additional analyses when the Dementia group was compared with the MCI group (Supplementary Table S4).

**Table 3:** Genotype and allele frequencies of the studied polymorphisms in the Control, MCI, and Dementia groups.

Gene	SNP	Genotype/Allele	Control (*n* = 84)	MCI (*n* = 94)	Dementia (*n* = 102)	MCI vs. Control χ^2^ (*p*)	Dem vs. Control χ^2^ (*p*)
*TOMM40*	rs2075650	AA, *n* (%)	49 (59.0)	57 (61.3)	52 (65.8)	χ^2^ = 0.20, *p* = 0.907	χ^2^ = 1.43, *p* = 0.489
		AG, *n* (%)	31 (37.3)	32 (34.4)	26 (32.9)		
		GG, *n* (%)	3 (3.6)	4 (4.3)	1 (1.3)		
		HWE χ^2^ (*p*)	0.51 (0.777)	0.03 (0.983)	1.31 (0.521)		
		Call rate (%)	98.8	98.8	70.8		
		Allele A, *n* (%)	129 (77.7)	146 (78.5)	130 (82.3)	χ^2^ = 0.03, *p* = 0.859	χ^2^ = 2.86, *p* = 0.239
		Allele G, *n* (%)	37 (22.3)	40 (21.5)	28 (17.7)		
*SORL1*	rs2070045	TT, *n* (%)	28 (39.4)	45 (51.7)	51 (51.5)	χ^2^ = 4.38, *p* = 0.112	χ^2^ = 2.47, *p* = 0.291
		TG, *n* (%)	37 (52.1)	31 (35.6)	42 (42.4)		
		GG, *n* (%)	6 (8.5)	11 (12.6)	6 (6.1)		
		HWE χ^2^ (*p*)	1.66 (0.436)	2.20 (0.333)	0.48 (0.788)		
		Call rate (%)	84.5	92.6	97.1		
		Allele T, *n* (%)	93 (65.5)	121 (69.5)	144 (72.7)	χ^2^ = 0.59, *p* = 0.444	χ^2^ = 2.05, *p* = 0.152
		Allele G, *n* (%)	49 (34.5)	53 (30.5)	54 (27.3)		
*SORL1*	rs3824968	TT, *n* (%)	28 (34.1)	37 (39.8)	36 (40.9)	χ^2^ = 3.46, *p* = 0.177	χ^2^ = 1.51, *p* = 0.469
		TA, *n* (%)	45 (54.9)	39 (41.9)	40 (45.5)		
		AA, *n* (%)	9 (11.0)	17 (18.3)	12 (13.6)		
		HWE χ^2^ (*p*)	2.09 (0.351)	1.35 (0.509)	0.04 (0.986)		
		Call rate (%)	97.6	98.9	86.3		
		Allele T, *n* (%)	101 (61.6)	113 (60.8)	112 (63.6)	χ^2^ = 0.03, *p* = 0.873	χ^2^ = 0.15, *p* = 0.696
		Allele A, *n* (%)	63 (38.4)	73 (39.2)	64 (36.4)		
*SORL1*	rs2282649	CC, *n* (%)	24 (32.4)	33 (37.9)	39 (38.6)	χ^2^ = 3.78, *p* = 0.151	χ^2^ = 1.42, *p* = 0.490
		CT, *n* (%)	44 (59.5)	40 (46.0)	51 (50.5)		
		TT, *n* (%)	6 (8.1)	14 (16.1)	11 (10.9)		
		HWE χ^2^ (*p*)	5.15 (0.076)	0.10 (0.950)	0.89 (0.640)		
		Call rate (%)	88.1	92.6	99.0		
		Allele C, *n* (%)	92 (62.2)	106 (60.9)	129 (63.9)	χ^2^ = 0.05, *p* = 0.819	χ^2^ = 0.11, *p* = 0.326
		Allele T, *n* (%)	56 (37.8)	68 (39.1)	73 (36.1)		
*SORL1* *	rs1010159	TT, *n* (%)	17 (23.6)	26 (28.9)	31 (31.3)	χ^2^ = 4.46, *p* = 0.107	χ^2^ = 2.37, *p* = 0.306
		TC, *n* (%)	47 (65.3)	45 (50.0)	53 (53.5)		
		CC, *n* (%)	8 (11.1)	19 (21.1)	15 (15.2)		
		HWE χ^2^ (*p*)	**7.66 (0.022)**	0.003 (0.998)	0.98 (0.645)		
		Call rate (%)	85.7	95.7	97.1		
		Allele T, *n* (%)	81 (56.3)	97 (53.9)	115 (58.1)	χ^2^ = 0.18, *p* = 0.671	χ^2^ = 0.11, *p* = 0.735
		Allele C, *n* (%)	63 (43.8)	83 (46.1)	83 (41.9)		
*BIN1*	rs744373	AA, *n* (%)	26 (44.1)	34 (44.2)	55 (55.0)	χ^2^ = 3.34, *p* = 0.189	χ^2^ = 3.53, *p* = 0.171
		AG, *n* (%)	31 (52.5)	34 (44.2)	38 (38.0)		
		GG, *n* (%)	2 (3.4)	9 (11.6)	7 (7.0)		
		HWE χ^2^ (*p*)	3.96 (0.140)	0.01 (0.990)	0.02 (0.990)		
		Call rate (%)	70.2	81.9	98.0		
		Allele A, *n* (%)	83 (70.3)	102 (66.2)	148 (74.0)	χ^2^ = 0.52, *p* = 0.472	χ^2^ = 0.50, *p* = 0.749
		Allele G, *n* (%)	35 (29.7)	52 (33.8)	52 (26.0)		
*BIN1* *	rs7561528	GG, *n* (%)	28 (35.9)	39 (43.3)	57 (56.4)	χ^2^ = 2.00, *p* = 0.367	**χ^2^ = 11.89, *p* = 0.003**
		GA, *n* (%)	47 (60.3)	45 (50.0)	35 (34.7)		
		AA, *n* (%)	3 (3.8)	6 (6.7)	9 (8.9)		
		HWE χ^2^ (*p*)	**9.18 (0.010)**	2.17 (0.340)	1.11 (0.570)		
		Call rate (%)	92.9	95.7	99.0		
		Allele G, *n* (%)	103 (66.0)	123 (68.3)	149 (73.8)	χ^2^ = 0.20, *p* = 0.653	χ^2^ = 2.53, *p* = 0.112
		Allele A, *n* (%)	53 (34.0)	57 (31.7)	53 (26.2)		
*BIN1* *	rs10200967	CC, *n* (%)	25 (33.8)	37 (43.0)	56 (56.6)	χ^2^ = 2.82, *p* = 0.244	**χ^2^ = 13.90, *p* = 0.001**
		CT, *n* (%)	47 (63.5)	44 (51.2)	35 (35.4)		
		TT, *n* (%)	2 (2.7)	5 (5.8)	8 (8.1)		
		HWE χ^2^ (*p*)	**12.20 (0.002)**	3.03 (0.220)	1.00 (0.607)		
		Call rate (%)	88.1	91.5	97.1		
		Allele C, *n* (%)	97 (65.5)	118 (68.6)	147 (74.2)	χ^2^ = 0.34, *p* = 0.561	χ^2^ = 3.09, *p* = 0.079
		Allele T, *n* (%)	51 (34.5)	54 (31.4)	51 (25.8)		
*VDR*	rs1544410	CC, *n* (%)	39 (50.6)	40 (44.4)	49 (49.5)	χ^2^ = 0.77, *p* = 0.681	χ^2^ = 2.11, *p* = 0.348
		CT, *n* (%)	30 (39.0)	41 (45.6)	45 (45.5)		
		TT, *n* (%)	8 (10.4)	9 (10.0)	5 (5.1)		
		HWE χ^2^ (*p*)	0.38 (0.828)	0.10 (0.950)	1.75 (0.417)		
		Call rate (%)	91.7	95.7	97.1		
		Allele C, *n* (%)	108 (70.1)	121 (67.2)	143 (72.2)	χ^2^ = 0.33, *p* = 0.568	χ^2^ = 0.18, *p* = 0.667
		Allele T, *n* (%)	46 (29.9)	59 (32.8)	55 (27.8)		
*VDR*	rs7975232	AA, *n* (%)	24 (30.8)	21 (23.6)	17 (17.0)	χ^2^ = 1.64, *p* = 0.439	χ^2^ = 5.49, *p* = 0.064
		AC, *n* (%)	33 (42.3)	46 (51.7)	57 (57.0)		
		CC, *n* (%)	21 (26.9)	22 (24.7)	26 (26.0)		
		HWE χ^2^ (*p*)	1.82 (0.404)	0.10 (0.950)	2.23 (0.329)		
		Call rate (%)	92.9	94.7	98.0		
		Allele A, *n* (%)	81 (51.9)	88 (49.4)	91 (45.5)	χ^2^ = 0.21, *p* = 0.650	χ^2^ = 1.45, *p* = 0.229
		Allele C, *n* (%)	75 (48.1)	90 (50.6)	109 (54.5)		
*CLU*	rs9331888	CC, *n* (%)	27 (35.1)	40 (44.0)	40 (39.2)	χ^2^ = 2.28, *p* = 0.319	χ^2^ = 1.58, *p* = 0.453
		CG, *n* (%)	34 (44.2)	39 (42.9)	48 (47.1)		
		GG, *n* (%)	16 (20.8)	12 (13.2)	14 (13.7)		
		HWE χ^2^ (*p*)	0.75 (0.688)	0.26 (0.879)	0.004 (0.998)		
		Call rate (%)	91.7	96.8	100.0		
		Allele C, *n* (%)	88 (57.1)	119 (65.4)	128 (62.7)	χ^2^ = 2.40, *p* = 0.122	χ^2^ = 1.15, *p* = 0.283
		Allele G, *n* (%)	66 (42.9)	63 (34.6)	76 (37.3)		
*CLU*	rs11136000	CC, *n* (%)	32 (47.8)	32 (41.6)	39 (39.8)	χ^2^ = 3.87, *p* = 0.144	χ^2^ = 1.35, *p* = 0.510
		CT, *n* (%)	29 (43.3)	29 (37.7)	46 (46.9)		
		TT, *n* (%)	6 (9.0)	16 (20.8)	13 (13.3)		
		HWE χ^2^ (*p*)	0.25 (0.988)	3.49 (0.175)	0.01 (0.995)		
		Call rate (%)	79.8	81.9	96.1		
		Allele C, *n* (%)	93 (69.4)	93 (60.4)	124 (63.3)	χ^2^ = 2.54, *p* = 0.111	χ^2^ = 1.33, *p* = 0.248
		Allele T, *n* (%)	41 (30.6)	61 (39.6)	72 (36.7)		
*CR1*	rs6656401	GG, *n* (%)	42 (57.5)	51 (60.0)	53 (53.5)	χ^2^ = 1.35, *p* = 0.509	χ^2^ = 1.06, *p* = 0.590
		GA, *n* (%)	23 (31.5)	29 (34.1)	38 (38.4)		
		AA, *n* (%)	8 (11.0)	5 (5.9)	8 (8.1)		
		HWE χ^2^ (*p*)	2.78 (0.249)	0.10 (0.945)	0.10 (0.949)		
		Call rate (%)	86.9	90.4	97.1		
		Allele G, *n* (%)	107 (73.3)	131 (77.1)	144 (72.7)	χ^2^ = 0.60, *p* = 0.438	χ^2^ = 0.02, *p* = 0.908
		Allele A, *n* (%)	39 (26.7)	39 (22.9)	54 (27.3)		
*CR1*	rs1408077	CC, *n* (%)	47 (58.0)	57 (62.0)	47 (50.0)	χ^2^ = 1.24, *p* = 0.537	χ^2^ = 1.64, *p* = 0.440
		CA, *n* (%)	26 (32.1)	30 (32.6)	39 (41.5)		
		AA, *n* (%)	8 (9.9)	5 (5.4)	8 (8.5)		
		HWE χ^2^ (*p*)	2.19 (0.334)	0.16 (0.924)	0.001 (0.999)		
		Call rate (%)	96.4	97.9	92.2		
		Allele C, *n* (%)	120 (74.1)	144 (78.3)	133 (70.7)	χ^2^ = 0.84, *p* = 0.361	χ^2^ = 0.48, *p* = 0.488
		Allele A, *n* (%)	42 (25.9)	40 (21.7)	55 (29.3)		
*PICALM*	rs541458	TT, *n* (%)	30 (43.5)	46 (53.5)	48 (49.0)	χ^2^ = 2.32, *p* = 0.314	χ^2^ = 0.66, *p* = 0.720
		TC, *n* (%)	29 (42.0)	33 (38.4)	39 (39.8)		
		CC, *n* (%)	10 (14.5)	7 (8.1)	11 (11.2)		
		HWE χ^2^ (*p*)	0.47 (0.792)	0.10 (0.952)	0.51 (0.776)		
		Call rate (%)	82.1	91.5	96.1		
		Allele T, *n* (%)	89 (64.5)	125 (72.7)	135 (68.9)	χ^2^ = 2.40, *p* = 0.121	χ^2^ = 0.70, *p* = 0.401
		Allele C, *n* (%)	49 (35.5)	47 (27.3)	61 (31.1)		

* Significant deviation from the Hardy–Weinberg equilibrium observed in the Controls. Data require cautious interpretation.

**Table 4:** Logistic regression analyses of the studied polymorphisms demonstrating nominal significance for associations with MCI or dementia. The reference genotype is the first genotype listed in the comparison column.

Gene	SNP	Genetic Model	Genotype Comparison	OR (95% CI)	*p*	*q* (FDR)
**MCI vs. Control**						
*SORL1*	rs2070045	Overdominant	TG vs. TT + GG	0.524 (0.276–0.995)	0.048	0.623
*SORL1*	rs1010159	Overdominant	TC vs. TT + CC	0.526 (0.278–0.997)	0.049	0.623
*CLU*	rs11136000	Recessive	TT vs. CC + CT	2.782 (1.016–7.616)	0.046	0.623
**Dementia vs. Control**						
*BIN1*	rs7561528	Codominant	GA vs. GG	0.329 (0.164–0.659)	0.002	0.075
*BIN1*	rs7561528	Dominant	GA + AA vs. GG	0.389 (0.199–0.758)	0.006	0.150
*BIN1*	rs7561528	Overdominant	GA vs. GG + AA	0.318 (0.162–0.624)	0.001	0.050
*BIN1*	rs10200967	Codominant	CT vs. CC	0.289 (0.142–0.588)	0.001	0.050
*BIN1*	rs10200967	Dominant	CT + TT vs. CC	0.350 (0.177–0.694)	0.003	0.090
*BIN1*	rs10200967	Overdominant	CT vs. CC + TT	0.272 (0.136–0.544)	<0.001	0.050
*VDR*	rs1544410	Recessive	TT vs. CC + CT	0.259 (0.081–0.834)	0.024	0.514
*VDR*	rs7975232	Codominant	AC vs. AA	2.308 (1.018–5.234)	0.045	0.623

**Table 5:** Logistic regression analyses of the studied polymorphisms demonstrating nominal significance for associations with dementia in females.

Gene	SNP	Genetic Model	Genotype Comparison	OR (95% CI)	*p*	*q* (FDR)
*BIN1*	rs7561528	Codominant	GA vs. GG	0.282 (0.126–0.632)	0.002	0.075
*BIN1*	rs7561528	Dominant	GA + AA vs. GG	0.345 (0.162–0.736)	0.006	0.150
*BIN1*	rs7561528	Overdominant	GA vs. GG + AA	0.275 (0.125–0.606)	0.001	0.050
*BIN1*	rs10200967	Codominant	CT vs. CC	0.260 (0.114–0.590)	0.001	0.050
*BIN1*	rs10200967	Dominant	CT + TT vs. CC	0.325 (0.150–0.704)	0.004	0.120
*BIN1*	rs10200967	Overdominant	CT vs. CC + TT	0.246 (0.110–0.550)	0.001	0.050

## 4. Discussion

This study evaluated associations between 15 genetic polymorphisms in seven candidate genes (*TOMM40*, *SORL1*, *BIN1*, *VDR*, *CLU*, *CR1*, and *PICALM*) and the risk of MCI and dementia in a Russian population. Under the primary FDR correction (60 tests per contrast), only polymorphisms in *BIN1* (rs7561528 and rs10200967) remained significantly associated with dementia. Nominally significant associations were observed for *SORL1* and *CLU* with MCI, and *VDR* with dementia, but these did not survive FDR correction and should be considered exploratory.

### 4.1. BIN1 Associations

The main finding of our study is the association of *BIN1* rs7561528 and rs10200967 with reduced dementia risk. The minor alleles of these SNPs (A for rs7561528, T for rs10200967) were consistently associated with protection across multiple genetic models (codominant, dominant, overdominant), with ORs ranging from 0.27 to 0.39. These associations remained significant under the primary FDR correction and require replication.

*BIN1* encodes a multifunctional adaptor protein involved in clathrin-mediated endocytosis, membrane trafficking, apoptosis, inflammation, and calcium homeostasis [24]. The GWAS signal at *BIN1* was originally identified through the intronic variant rs744373, which we also analyzed but did not find significantly associated with dementia in our cohort. However, rs744373 has been consistently associated with tau pathology, with carriers of the risk allele showing increased tau-PET levels and worse memory performance [25].

The biological relevance of BIN1 in neurodegeneration is supported by multiple lines of evidence. First, BIN1 directly interacts with tau protein and modulates its phosphorylation and aggregation [39]. Overexpression of *BIN1* promotes tau pathology, while its knockdown reduces tau levels [41]. Second, *BIN1* is predominantly expressed in oligodendrocytes, where it regulates myelination and white matter integrity [24]. Disruption of BIN1 function in oligodendrocytes may contribute to the white matter abnormalities observed in AD and dementia [42]. Third, BIN1 is involved in APP trafficking and endocytosis, and its deficiency has been shown to modulate amyloid-β production [43]. Thus, BIN1 may influence dementia risk through multiple convergent pathways: tau pathology, myelin integrity, and amyloid processing. These mechanistic explanations are speculative and grounded in the prior literature rather than directly demonstrated by our data.

Our findings for rs7561528 and rs10200967 extend the known role of *BIN1* in dementia risk. Notably, the protective effect of the minor alleles observed in our cohort contrasts with the typical risk allele designation reported in some European GWAS. This may reflect population-specific linkage disequilibrium patterns, where these SNPs tag different functional variants than in other populations [44]. Alternatively, it may be due to gene-environment interactions specific to the Russian population. This finding underscores the importance of population-specific genetic studies and the limitations of extrapolating GWAS results from one ethnicity to another.

The consistency of the *BIN1* effect across three genetic models strengthens the credibility of this finding. The magnitude of the effect (OR ≈ 0.3) is notably larger than the modest effects typically observed in GWAS for complex traits (OR ≈ 1.15–1.20) [28]. While this could reflect a genuinely strong protective effect in this population, it may also be influenced by the relatively small sample size and should be interpreted with caution. Allele frequencies and linkage disequilibrium patterns at the *BIN1* locus differ across populations, and the tag SNPs analysed here may capture different functional variants in the Russian population. In addition, *BIN1* rs7561528 and rs10200967 deviated from the Hardy–Weinberg equilibrium in the Controls, which may have influenced effect estimates. The Control group showed deviation from HWE for these SNPs, which may reflect genotyping artefact or population stratification. Although the concordance rate was 100%, cluster separation was acceptable, and the BIN1 associations persisted in female-only analysis, these findings should be interpreted with caution pending replication in larger, independent cohorts. Nevertheless, replication in larger independent cohorts is essential to confirm this finding.

It is important to note that the statistical significance of the BIN1 findings depends on the scope of the multiple-testing correction. Under the primary analysis, which included 15 SNPs under four genetic models in two main contrasts (MCI vs. Control and Dementia vs. Control; 60 tests per contrast), both BIN1 rs7561528 and rs10200967 remained significant after Benjamini–Hochberg FDR correction (*q* < 0.05). When the correction was extended to all 150 tests, including exploratory sensitivity analyses such as the female-only comparison and the Dementia vs. MCI contrast, the associations were at the threshold of significance (*q* = 0.050).

We consider the 150-test correction to be overly conservative for the primary hypothesis, as it penalizes the analysis for exploratory comparisons that were not part of the pre-specified primary aim. Such an approach substantially increases the risk of type II error, potentially discarding true associations. This concern is particularly relevant in candidate-gene studies with limited sample sizes, where excessive correction can obscure biologically plausible signals.

Nevertheless, we acknowledge that the BIN1 associations do not survive the most stringent correction, and we therefore present them as the primary finding while emphasizing the need for replication in larger, independent cohorts.

### 4.2. SORL1 Associations

Heterozygous genotypes of *SORL1* rs2070045 (TG) and rs1010159 (TC) were associated with reduced MCI risk under the overdominant model, while the *CLU* rs11136000 TT genotype was associated with increased MCI risk under the recessive model. However, these associations were only nominally significant and did not survive FDR correction.

Despite the lack of statistical robustness, these findings are biologically plausible and consistent with the previous literature. Gao et al. [23] demonstrated that *SORL1* rs2070045 was associated with amnestic MCI in a northern Han Chinese population, and Piscopo et al. [22] reported that *SORL1* variants, including rs1010159, were associated with conversion from MCI to AD. *SORL1* encodes a receptor that directs APP trafficking; its deficiency shifts APP toward β-secretase cleavage, increasing amyloid-β production [21]. This mechanism is speculative and not directly tested in our study. The protective effect of heterozygous genotypes may reflect a dosage-dependent mechanism. Intermediate SORL1 expression may optimize APP trafficking, whereas homozygous genotypes disrupt this balance.

### 4.3. CLU Associations

Similarly, *CLU* encodes a chaperone protein involved in amyloid-β clearance, complement regulation, and neuroinflammation [12]. The *CLU* locus is well established as an AD risk locus in GWAS [9], and elevated plasma CLU levels have been observed in MCI patients [14]. However, our findings partially contradict Cai et al. [16], who reported that the TT genotype of rs11136000 was associated with reduced MCI risk in type 2 diabetic patients. This discrepancy may reflect population differences, interaction with metabolic factors, or the presence of other genetic modifiers.

Given the nominal nature of these associations, we emphasize that they should be considered hypothesis-generating rather than confirmatory. Future studies with larger sample sizes are required to determine whether these associations represent true effects or false positives.

### 4.4. VDR Associations

The *VDR* polymorphisms rs1544410 and rs7975232 showed distinct patterns of association with dementia. The TT genotype of rs1544410 was associated with reduced dementia risk, while the AC genotype of rs7975232 was associated with increased risk. However, neither association survived FDR correction.

The VDR is widely expressed in the brain, including the substantia nigra, cortex, thalamus, and hypothalamus [29]. Importantly, rs1544410 and rs7975232 are functional regulatory variants: the B allele (C at rs1544410) and A allele (A at rs7975232) are associated with greater VDR mRNA stability and protein expression, while the b (T) and a (C) alleles are associated with reduced expression [32,33].

Our findings align with this functional framework: the less active TT genotype of rs1544410 (bb) was protective against dementia, while the heterozygous AC genotype (Aa) was associated with increased risk. However, because these associations did not survive FDR correction, they should be interpreted with caution. However, the direction of effect remains debated. This may reflect population-specific linkage disequilibrium and the role of vitamin D status as an environmental modifier.

### 4.5. Negative Findings

No significant associations were observed for *TOMM40* rs2075650, *SORL1* rs2282649 and rs3824968, *BIN1* rs744373, *CLU* rs9331888, *CR1* rs6656401 and rs1408077, or *PICALM* rs541458, even at the nominal level. Several explanations may account for these negative findings.

### 4.6. Limitations and Future Directions

First, the relatively small sample size (*n* = 280, with 102 dementia cases and 94 MCI cases) limits statistical power to detect modest genetic effects. For a SNP with a minor allele frequency of 0.2 and an OR of 1.5, the required sample size for 80% power is approximately 500–700 cases. Our study was underpowered for such effect sizes.

Second, the selected variants are predominantly intronic tag SNPs that mark associated chromosomal regions but are not themselves functional. The causal variants responsible for these associations may differ across populations due to linkage disequilibrium structure [44].

Third, the absence of *APOE* genotyping in our panel, the strongest genetic risk factor for dementia, represents a limitation. The effects of other loci may be masked or modified by APOE status, and future studies should include *APOE* to assess potential interactions. The prevalence and effect size of the APOE ε4 allele in Russian populations have been characterized in our previous study of 867 individuals from the European part of Russia, in which APOE ε4 heterozygosity was associated with an OR of 1.81 and homozygosity with an OR of 7.98 for dementia [45]. This is comparable to or slightly lower than in other European populations, suggesting that the absence of APOE genotyping in our panel may have a similar impact on risk stratification as in other European cohorts [45].

Fourth, the Control group was predominantly female (95%), reflecting the general pattern of health-seeking behaviour in Russia, where older women are more likely to attend routine medical assessments and to participate in clinical research than men of the same age. Although the BIN1 associations persisted in female-only analysis, this imbalance remains a limitation, and the sex-adjusted odds ratios should be interpreted with caution.

Fifth, three SNPs (SORL1 rs1010159, BIN1 rs7561528, and BIN1 rs10200967) deviated from Hardy–Weinberg equilibrium in the Control group, which may reflect genotyping artefact or population stratification. Although the concordance rate was 100% and cluster separation was acceptable, these findings should be interpreted with caution.

Sixth, the Dementia group was aetiologically heterogeneous: the majority of patients had mixed dementia (ICD-10: F02.8), and the specific aetiology could not be determined clinically due to the severity of cognitive impairment and the absence of biomarker confirmation.

Our findings suggest that *BIN1* genotyping could contribute to risk stratification for dementia in the Russian population. However, the clinical utility of the panel currently rests primarily on the *BIN1* associations, as the other findings require replication.

The differential associations of *VDR* haplotypes, if confirmed in larger studies, may provide guidance for personalized vitamin D supplementation strategies. For MCI, the exploratory findings with *SORL1* and *CLU* require further investigation before any clinical application.

## 5. Conclusions

This study identified associations between genetic polymorphisms in *BIN1*, *SORL1*, *CLU*, and *VDR* and the risk of MCI and dementia in a Russian population. Under the primary FDR correction (60 tests per contrast), only BIN1 rs7561528 and rs10200967 remained significantly associated with reduced odds of dementia. Under the more conservative 150-test correction, these associations were at the threshold of significance (*q* = 0.050), representing the hypothesis-generating finding of our study. Nominally significant associations were observed for *SORL1* rs2070045 and rs1010159 and *CLU* rs11136000 with MCI, as well as for *VDR* rs1544410 and rs7975232 with dementia, but these did not survive FDR correction and should be considered exploratory, requiring replication in larger independent cohorts.

The *BIN1* polymorphisms identified in this study may contribute to genetic risk assessment for dementia in the Russian population. These findings should be viewed as preliminary, requiring replication in larger, independent cohorts.

## Figures and Tables

**Table 1:** Candidate genes and selected polymorphisms.

Gene	Protein/Function	Selected SNPs	Chromosome	Effect Allele	Other Allele
*TOMM40*	Translocase of outer mitochondrial membrane 40; involved in mitochondrial protein import and energy metabolism; associated with AD and cognitive aging	rs2075650	19	A	G
*SORL1*	Sortilin-related receptor 1; directs APP trafficking and recycling; deficiency shifts APP toward β-secretase cleavage, increasing amyloid production; associated with AD and MCI	rs2070045 rs2282649 rs1010159 rs3824968	11 11 11 11	T C T T	G T C A
*BIN1*	Bridging integrator 1; involved in protein trafficking, apoptosis, inflammation, and calcium homeostasis; associated with tau accumulation and AD risk; second most significant GWAS locus	rs744373 rs7561528 rs10200967	2 2 2	A G C	G A T
*VDR*	Vitamin D receptor; widely expressed in the brain; regulates neurotransmission (acetylcholine, dopamine, serotonin, GABA), neuroinflammation, and neuroprotection; polymorphisms affect mRNA stability and protein expression	rs1544410 rs7975232	12 12	C A	T C
*CLU*	Clusterin (apolipoprotein J); chaperone protein involved in amyloid-β clearance, complement regulation, and neuroinflammation; identified as AD risk locus in GWAS	rs9331888 rs11136000	8 8	C C	G T
*CR1*	Complement receptor type 1; mediates complement activation, immune complex clearance, and complement-mediated synaptic pruning; associated with AD risk	rs6656401 rs1408077	1 1	G C	A A
*PICALM*	Phosphatidylinositol-binding clathrin assembly protein; involved in clathrin-mediated endocytosis and APP trafficking; GWAS-confirmed AD risk locus	rs541458	11	T	C

**Table 2:** Demographic and clinical characteristics of the study population.

Characteristic	Control (*n* = 84)	MCI (*n* = 94)	Dementia (*n* = 102)	Statistic	*p*
Age, years, median [IQR]	69 [65; 73]	70 [66; 73]	67 [61; 73]	H = 9.77	0.0076
Sex, male/female	4/80	8/86	54/48	χ^2^ = 70.2	<0.0001
MoCA score, median [IQR]	27 [26; 28]	22 [21; 23]	9 [6; 13]	H = 176.7	<0.0001

## Data Availability

The data presented in this study are available from the corresponding author upon request.

## Supplementary Materials

The following supporting information can be downloaded at https://www.mdpi.com/article/10.3390/diseases14100368/s1. Table S1: Full results of association analysis for all 15 SNPs under four genetic models; Table S2. Genotype and allele frequencies of the studied polymorphisms in the Control, MCI, and Dementia groups in females only; Table S3. Full results of association analysis for all 15 SNPs under four genetic models in females with MCI or dementia compared with controls; Table S4. Full results of association analysis for all 15 SNPs under four genetic models in females with dementia compared with MCI; Full results of the association analysis for all 15 SNPs under four genetic models.

## Abbreviations

The following abbreviations are used in this manuscript:

AD	Alzheimer’s disease
ADGC	Alzheimer’s Disease Genetics Consortium
APOE	Apolipoprotein E
APP	Amyloid precursor protein
BIN1	Bridging integrator 1
CLU	Clusterin
CR1	Complement receptor 1
FDR	False discovery rate
GABA	Gamma-aminobutyric acid
GWAS	Genome-wide association studies
HWE	Hardy–Weinberg equilibrium
ICD-10	International Statistical Classification of Diseases and Related Health Problems, 10th Revision
MCI	Mild cognitive impairment
MoCA	Montreal Cognitive Assessment
PICALM	Phosphatidylinositol-binding clathrin assembly protein
SNP	Single nucleotide polymorphism
SORL1	Sortilin-related receptor 1
TOMM40	Translocase of outer mitochondrial membrane 40
VDR	Vitamin D receptor
