# Supplementary material for: Genetic Polymorphisms in SORL1, CLU, BIN1, and VDR Are Associated with Mild Cognitive Impairment and Dementia in a Russian Population
Source: Diseases. 2026 Oct 3;14(10):368. doi: 10.3390/diseases14100368 (PMC13703006; doi:10.3390/diseases14100368)
Supplement: Supplementary file 1 [file diseases-14-00368-s001.zip › Supplementary Table S1.pdf]

Supplementary Table S1. Full results of association analysis for all 15 SNPs under four genetic models.

Gene	SNP	Comparison	Model	Genotype	OR (95% CI)	p	q (FDR)
TOMM40	rs2075650	MCI vs Control	Codominant	AG vs AA	0.867 (0.463–1.624)	0.657	0.782
				GG vs AA	1.083 (0.230–5.100)	0.920	0.932
			Dominant	AG+GG vs AA	0.886 (0.483–1.627)	0.697	0.805
			Recessive	GG vs AA+AG	1.142 (0.247–5.283)	0.865	0.901
			Overdominant	AG vs AA+GG	0.864 (0.465–1.605)	0.643	0.772
		Dementia vs Control	Codominant	AG vs AA	0.880 (0.426–1.817)	0.730	0.805
				GG vs AA	0.269 (0.023–3.141)	0.295	0.626
			Dominant	AG+GG vs AA	0.810 (0.399–1.647)	0.561	0.725
			Recessive	GG vs AA+AG	0.283 (0.025–3.252)	0.311	0.626
			Overdominant	AG vs AA+GG	0.926 (0.451–1.901)	0.834	0.896
SORL1	rs2070045	MCI vs Control	Codominant	TG vs TT	0.534 (0.272–1.046)	0.068	0.623
				GG vs TT	1.106 (0.367–3.336)	0.858	0.900
			Dominant	TG+GG vs TT	0.617 (0.327–1.167)	0.138	0.623
			Recessive	GG vs TT+TG	1.499 (0.523–4.293)	0.451	0.657
			Overdominant	TG vs TT+GG	**0.524 (0.276–0.995)**	**0.048**	0.623
		Dementia vs Control	Codominant	TG vs TT	0.719 (0.359–1.443)	0.354	0.626
				GG vs TT	0.493 (0.129–1.890)	0.302	0.626
			Dominant	TG+GG vs TT	0.680 (0.347–1.333)	0.262	0.626
			Recessive	GG vs TT+TG	0.587 (0.161–2.145)	0.421	0.638
			Overdominant	TG vs TT+GG	0.792 (0.405–1.547)	0.494	0.693
SORL1	rs3824968	MCI vs Control	Codominant	TA vs TT	0.653 (0.340–1.254)	0.200	0.623
				AA vs TT	1.479 (0.573–3.818)	0.419	0.638
			Dominant	TA+AA vs TT	0.787 (0.424–1.461)	0.448	0.657
			Recessive	AA vs TT+TA	1.882 (0.786–4.503)	0.156	0.623
			Overdominant	TA vs TT+AA	0.586 (0.321–1.068)	0.081	0.623
		Dementia vs Control	Codominant	TA vs TT	0.731 (0.356–1.502)	0.394	0.635
				AA vs TT	1.300 (0.443–3.818)	0.633	0.772
			Dominant	TA+AA vs TT	0.823 (0.414–1.634)	0.578	0.735
			Recessive	AA vs TT+TA	1.557 (0.575–4.215)	0.383	0.631
			Overdominant	TA vs TT+AA	0.684 (0.352–1.331)	0.264	0.626
SORL1	rs2282649	MCI vs Control	Codominant	CT vs CC	0.651 (0.330–1.285)	0.216	0.623

Gene	SNP	Comparison	Model	Genotype	OR (95% CI)	p	q (FDR)
				TT vs CC	1.735 (0.580–5.187)	0.324	0.626
			Dominant	CT+TT vs CC	0.778 (0.405–1.495)	0.451	0.657
			Recessive	TT vs CC+CT	2.241 (0.812–6.188)	0.119	0.623
			Overdominant	CT vs CC+TT	0.569 (0.303–1.067)	0.079	0.623
		Dementia vs Control	Codominant	CT vs CC	0.751 (0.370–1.523)	0.428	0.642
				TT vs CC	1.252 (0.375–4.178)	0.715	0.805
			Dominant	CT+TT vs CC	0.811 (0.408–1.609)	0.549	0.716
			Recessive	TT vs CC+CT	1.491 (0.483–4.598)	0.487	0.689
			Overdominant	CT vs CC+TT	0.718 (0.371–1.390)	0.325	0.626
SORL1	rs1010159	MCI vs Control	Codominant	TC vs TT	0.625 (0.299–1.306)	0.212	0.623
				CC vs TT	1.597 (0.570–4.475)	0.374	0.631
			Dominant	TC+CC vs TT	0.765 (0.376–1.558)	0.461	0.665
			Recessive	CC vs TT+TC	2.203 (0.900–5.394)	0.084	0.623
			Overdominant	TC vs TT+CC	**0.526 (0.278–0.997)**	**0.049**	0.623
		Dementia vs Control	Codominant	TC vs TT	0.652 (0.300–1.417)	0.280	0.626
				CC vs TT	1.194 (0.391–3.643)	0.755	0.827
			Dominant	TC+CC vs TT	0.734 (0.346–1.556)	0.419	0.638
			Recessive	CC vs TT+TC	1.606 (0.603–4.281)	0.343	0.626
			Overdominant	TC vs TT+CC	0.612 (0.309–1.213)	0.159	0.623
BIN1	rs744373	MCI vs Control	Codominant	AG vs AA	0.845 (0.416–1.713)	0.640	0.772
				GG vs AA	3.591 (0.712–18.125)	0.122	0.623
			Dominant	AG+GG vs AA	1.009 (0.509–2.000)	0.980	0.987
			Recessive	GG vs AA+AG	3.926 (0.812–18.978)	0.089	0.623
			Overdominant	AG vs AA+GG	0.715 (0.362–1.416)	0.336	0.626
		Dementia vs Control	Codominant	AG vs AA	0.565 (0.274–1.165)	0.122	0.623
				GG vs AA	1.758 (0.317–9.755)	0.519	0.705
			Dominant	AG+GG vs AA	0.638 (0.316–1.286)	0.209	0.623
			Recessive	GG vs AA+AG	2.305 (0.431–12.330)	0.329	0.626
			Overdominant	AG vs AA+GG	0.535 (0.264–1.085)	0.083	0.623
BIN1	rs7561528	MCI vs Control	Codominant	GA vs GG	0.703 (0.372–1.329)	0.278	0.626
				AA vs GG	1.525 (0.350–6.651)	0.575	0.735
			Dominant	GA+AA vs GG	0.753 (0.403–1.408)	0.374	0.631

Gene	SNP	Comparison	Model	Genotype	OR (95% CI)	p	q (FDR)
			Recessive	AA vs GG+GA	1.885 (0.454–7.832)	0.383	0.631
			Overdominant	GA vs GG+AA	0.669 (0.361–1.237)	0.200	0.623
		Dementia vs Control	Codominant	GA vs GG	**0.329 (0.164–0.659)**	**0.002**	0.075
				AA vs GG	1.323 (0.304–5.762)	0.709	0.805
			Dominant	GA+AA vs GG	**0.389 (0.199–0.758)**	**0.006**	0.150
			Recessive	AA vs GG+GA	2.310 (0.556–9.601)	0.249	0.626
			Overdominant	GA vs GG+AA	**0.318 (0.162–0.624)**	**0.001**	0.050
BIN1	rs10200967	MCI vs Control	Codominant	CT vs CC	0.642 (0.333–1.237)	0.186	0.623
				TT vs CC	1.793 (0.321–10.016)	0.506	0.696
			Dominant	CT+TT vs CC	0.691 (0.362–1.318)	0.262	0.626
			Recessive	TT vs CC+CT	2.351 (0.441–12.537)	0.317	0.626
			Overdominant	CT vs CC+TT	0.606 (0.321–1.146)	0.123	0.623
		Dementia vs Control	Codominant	CT vs CC	**0.289 (0.142–0.588)**	**0.001**	0.050
				TT vs CC	1.781 (0.328–9.667)	0.504	0.696
			Dominant	CT+TT vs CC	**0.350 (0.177–0.694)**	**0.003**	0.090
			Recessive	TT vs CC+CT	3.358 (0.647–17.434)	0.149	0.623
			Overdominant	CT vs CC+TT	**0.272 (0.136–0.544)**	**<0.001**	0.050
VDR	rs1544410	MCI vs Control	Codominant	CT vs CC	1.336 (0.700–2.551)	0.380	0.631
				TT vs CC	0.639 (0.248–1.651)	0.355	0.626
			Dominant	CT+TT vs CC	1.114 (0.611–2.030)	0.726	0.805
			Recessive	TT vs CC+CT	0.558 (0.227–1.371)	0.203	0.623
			Overdominant	CT vs CC+TT	1.476 (0.800–2.722)	0.213	0.623
		Dementia vs Control	Codominant	CT vs CC	1.428 (0.718–2.840)	0.310	0.626
				TT vs CC	0.308 (0.092–1.037)	0.057	0.623
			Dominant	CT+TT vs CC	1.065 (0.559–2.031)	0.848	0.896
			Recessive	TT vs CC+CT	**0.259 (0.081–0.834)**	**0.024**	0.514
			Overdominant	CT vs CC+TT	1.733 (0.898–3.344)	0.101	0.623
VDR	rs7975232	MCI vs Control	Codominant	AC vs AA	1.629 (0.778–3.412)	0.196	0.623
				CC vs AA	1.196 (0.517–2.767)	0.676	0.796
			Dominant	AC+CC vs AA	1.460 (0.734–2.904)	0.281	0.626
			Recessive	CC vs AA+AC	0.877 (0.437–1.760)	0.713	0.805
			Overdominant	AC vs AA+CC	1.492 (0.808–2.758)	0.201	0.623

Gene	SNP	Comparison	Model	Genotype	OR (95% CI)	p	q (FDR)
		Dementia vs Control	Codominant	AC vs AA	**2.308 (1.018–5.234)**	**0.045**	0.623
				CC vs AA	1.476 (0.584–3.733)	0.410	0.638
			Dominant	AC+CC vs AA	1.979 (0.916–4.273)	0.082	0.623
			Recessive	CC vs AA+AC	0.838 (0.399–1.760)	0.640	0.772
			Overdominant	AC vs AA+CC	1.868 (0.970–3.598)	0.062	0.623
CLU	rs9331888	MCI vs Control	Codominant	CG vs CC	0.753 (0.384–1.475)	0.408	0.638
				GG vs CC	0.470 (0.191–1.156)	0.100	0.623
			Dominant	CG+GG vs CC	0.663 (0.354–1.242)	0.199	0.623
			Recessive	GG vs CC+CG	0.547 (0.240–1.247)	0.151	0.623
			Overdominant	CG vs CC+GG	0.941 (0.510–1.739)	0.847	0.896
		Dementia vs Control	Codominant	CG vs CC	0.860 (0.422–1.754)	0.679	0.796
				GG vs CC	0.443 (0.167–1.178)	0.103	0.623
			Dominant	CG+GG vs CC	0.727 (0.372–1.418)	0.350	0.626
			Recessive	GG vs CC+CG	0.480 (0.196–1.176)	0.108	0.623
			Overdominant	CG vs CC+GG	1.089 (0.570–2.083)	0.796	0.865
CLU	rs11136000	MCI vs Control	Codominant	CT vs CC	1.037 (0.508–2.118)	0.920	0.932
				TT vs CC	2.835 (0.979–8.216)	0.055	0.623
			Dominant	CT+TT vs CC	1.340 (0.690–2.602)	0.387	0.631
			Recessive	TT vs CC+CT	**2.782 (1.016–7.616)**	**0.046**	0.623
			Overdominant	CT vs CC+TT	0.807 (0.413–1.576)	0.530	0.705
		Dementia vs Control	Codominant	CT vs CC	1.434 (0.693–2.966)	0.331	0.626
				TT vs CC	2.132 (0.676–6.728)	0.196	0.623
			Dominant	CT+TT vs CC	1.558 (0.781–3.110)	0.208	0.623
			Recessive	TT vs CC+CT	1.768 (0.597–5.233)	0.303	0.626
			Overdominant	CT vs CC+TT	1.210 (0.611–2.396)	0.585	0.737
CR1	rs6656401	MCI vs Control	Codominant	GA vs GG	0.999 (0.500–1.997)	0.998	0.998
				AA vs GG	0.490 (0.148–1.619)	0.242	0.626
			Dominant	GA+AA vs GG	0.894 (0.473–1.690)	0.729	0.805
			Recessive	AA vs GG+GA	0.484 (0.150–1.559)	0.224	0.626
			Overdominant	GA vs GG+AA	1.127 (0.578–2.199)	0.725	0.805
		Dementia vs Control	Codominant	GA vs GG	1.261 (0.614–2.588)	0.528	0.705
				AA vs GG	0.556 (0.162–1.904)	0.350	0.626

Gene	SNP	Comparison	Model	Genotype	OR (95% CI)	p	q (FDR)
			Dominant	GA+AA vs GG	1.072 (0.549–2.095)	0.838	0.896
			Recessive	AA vs GG+GA	0.508 (0.153–1.688)	0.269	0.626
			Overdominant	GA vs GG+AA	1.352 (0.671–2.724)	0.399	0.637
CR1	rs1408077	MCI vs Control	Codominant	CA vs CC	0.961 (0.500–1.847)	0.905	0.932
				AA vs CC	0.510 (0.155–1.673)	0.266	0.626
			Dominant	CA+AA vs CC	0.856 (0.464–1.577)	0.618	0.772
			Recessive	AA vs CC+CA	0.517 (0.161–1.659)	0.267	0.626
			Overdominant	CA vs CC+AA	1.034 (0.545–1.961)	0.918	0.932
		Dementia vs Control	Codominant	CA vs CC	1.430 (0.709–2.886)	0.318	0.626
				AA vs CC	0.629 (0.180–2.200)	0.469	0.670
			Dominant	CA+AA vs CC	1.235 (0.638–2.389)	0.531	0.705
			Recessive	AA vs CC+CA	0.527 (0.172–1.608)	0.260	0.626
			Overdominant	CA vs CC+AA	1.503 (0.758–2.978)	0.243	0.626
PICALM	rs541458	MCI vs Control	Codominant	TC vs TT	0.736 (0.373–1.453)	0.377	0.631
				CC vs TT	0.445 (0.152–1.302)	0.139	0.623
			Dominant	TC+CC vs TT	0.661 (0.349–1.253)	0.205	0.623
			Recessive	CC vs TT+TC	0.512 (0.183–1.428)	0.201	0.623
			Overdominant	TC vs TT+CC	0.856 (0.448–1.636)	0.638	0.772
		Dementia vs Control	Codominant	TC vs TT	0.698 (0.339–1.438)	0.329	0.626
				CC vs TT	0.496 (0.167–1.470)	0.206	0.623
			Dominant	TC+CC vs TT	0.645 (0.328–1.270)	0.205	0.623
			Recessive	CC vs TT+TC	0.586 (0.209–1.644)	0.310	0.626
			Overdominant	TC vs TT+CC	0.806 (0.406–1.599)	0.537	0.707
