# Supplementary material for: Genetic Polymorphisms in SORL1, CLU, BIN1, and VDR Are Associated with Mild Cognitive Impairment and Dementia in a Russian Population
Source: Diseases. 2026 Oct 3;14(10):368. doi: 10.3390/diseases14100368 (PMC13703006; doi:10.3390/diseases14100368)
Supplement: Supplementary file 1 [file diseases-14-00368-s001.zip › Supplementary Tables S2-S4.pdf]

Supplementary Table S2**.** Genotype and allele frequencies of the studied polymorphisms in the Control, MCI, and Dementia groups in females only

Gene	SNP	Genotype/Allele	Control (n=80)	MCI (n=86)	Dementia (n=48)	MCI vs Control χ^2 (p)	Dem vs Control χ^2 (p)
TOMM40	rs2075650	AA, n (%)	46 (58.2)	52 (61.2)	22 (64.7)	χ^2 = 0.31, p = 0.857	χ^2 = 0.42, p = 0.810
		AG, n (%)	30 (38.0)	29 (34.1)	11 (32.4)		
		GG, n (%)	3 (3.8)	4 (4.7)	1 (2.9)		
		HWE χ^2 (p)	0.50 (0.781)	0.00 (0.999)	0.07 (0.965)		
		Call rate (%)	98.8	98.8	70.8		
		Allele A, n (%)	122 (77.2)	133 (78.2)	55 (80.9)	χ^2 = 0.05, p = 0.824	χ^2 = 0.38, p = 0.539
		Allele G, n (%)	36 (22.8)	37 (21.8)	13 (19.1)		
SORL1	rs2070045	TT, n (%)	26 (38.8)	41 (51.9)	21 (46.7)	χ^2 = 3.56, p = 0.169	χ^2 = 0.74, p = 0.691
		TG, n (%)	35 (52.2)	29 (36.7)	21 (46.7)		
		GG, n (%)	6 (9.0)	9 (11.4)	3 (6.7)		
		HWE χ^2 (p)	1.45 (0.485)	1.17 (0.557)	0.56 (0.758)		
		Call rate (%)	83.8	91.9	93.8		
		Allele T, n (%)	87 (64.9)	111 (70.3)	63 (70.0)	χ^2 = 0.94, p = 0.332	χ^2 = 0.63, p = 0.429
		Allele G, n (%)	47 (35.1)	47 (29.7)	27 (30.0)		

Gene	SNP	Genotype/Allele	Control (n=80)	MCI (n=86)	Dementia (n=48)	MCI vs Control χ^2 (p)	Dem vs Control χ^2 (p)
		TT, n (%)	27 (34.6)	34 (40.0)	14 (35.0)	$\chi^2 = 3.10$, p = 0.212	$\chi^2 = 0.90$, p = 0.638
		TA, n (%)	42 (53.8)	35 (41.2)	19 (47.5)		
		AA, n (%)	9 (11.5)	16 (18.8)	7 (17.5)		
SORL1	rs3824968	HWE χ^2 (p)	1.47 (0.478)	1.61 (0.446)	0.02 (0.992)		
		Call rate (%)	97.5	98.8	83.3		
		Allele T, n (%)	96 (61.5)	103 (60.6)	47 (58.8)	$\chi^2 = 0.03$, p = 0.860	$\chi^2 = 0.17$, p = 0.678
		Allele A, n (%)	60 (38.5)	67 (39.4)	33 (41.3)		
		CC, n (%)	23 (32.9)	31 (39.2)	16 (34.0)	$\chi^2 = 3.71$, p = 0.157	$\chi^2 = 0.19$, p = 0.909
		CT, n (%)	41 (58.6)	35 (44.3)	26 (55.3)		
		TT, n (%)	6 (8.6)	13 (16.5)	5 (10.6)		
SORL1	rs2282649	HWE χ^2 (p)	4.20 (0.123)	0.34 (0.845)	1.37 (0.505)		
		Call rate (%)	87.5	91.9	97.9		
		Allele C, n (%)	87 (62.1)	97 (61.4)	58 (61.7)	$\chi^2 = 0.02$, p = 0.894	$\chi^2 = 0.00$, p = 0.946
		Allele T, n (%)	53 (37.9)	61 (38.6)	36 (38.3)		
SORL1	rs1010159	TT, n (%)	17 (25.0)	24 (29.3)	13 (28.3)	$\chi^2 = 3.88$, p = 0.144	$\chi^2 = 1.09$, p = 0.579

Gene	SNP	Genotype/Allele	Control (n=80)	MCI (n=86)	Dementia (n=48)	MCI vs Control χ^2 (p)	Dem vs Control χ^2 (p)
		TC, n (%)	43 (63.2)	40 (48.8)	25 (54.3)		
		CC, n (%)	8 (11.8)	18 (22.0)	8 (17.4)		
		HWE χ^2 (p)	5.61 (0.060)	0.03 (0.985)	0.46 (0.794)		
		Call rate (%)	85.0	95.3	95.8		
		Allele T, n (%)	77 (56.6)	88 (53.7)	51 (55.4)	χ^2 = 0.26, p = 0.608	χ^2 = 0.03, p = 0.860
		Allele C, n (%)	59 (43.4)	76 (46.3)	41 (44.6)		
BIN1	rs744373	AA, n (%)	25 (44.6)	30 (43.5)	26 (56.5)	χ^2 = 2.80, p = 0.247	χ^2 = 3.50, p = 0.174
		AG, n (%)	29 (51.8)	31 (44.9)	16 (34.8)		
		GG, n (%)	2 (3.6)	8 (11.6)	4 (8.7)		
		HWE χ^2 (p)	3.39 (0.184)	0.00 (0.999)	0.44 (0.802)		
		Call rate (%)	70.0	80.2	95.8		
		Allele A, n (%)	79 (70.5)	91 (65.9)	68 (73.9)	χ^2 = 0.60, p = 0.439	χ^2 = 0.29, p = 0.593
		Allele G, n (%)	33 (29.5)	47 (34.1)	24 (26.1)		
BIN1	rs7561528	GG, n (%)	28 (37.8)	35 (42.7)	30 (63.8)	χ^2 = 0.88, p = 0.644	**χ^2 = 10.80, p = 0.005**
		GA, n (%)	43 (58.1)	42 (51.2)	13 (27.7)		

Gene	SNP	Genotype/Allele	Control (n=80)	MCI (n=86)	Dementia (n=48)	MCI vs Control χ² (p)	Dem vs Control χ² (p)
		AA, n (%)	3 (4.1)	5 (6.1)	4 (8.5)		
		HWE χ² (p)	**7.20 (0.027)**	2.74 (0.255)	1.93 (0.382)		
		Call rate (%)	92.5	95.3	97.9		
		Allele G, n (%)	99 (66.9)	112 (68.3)	73 (77.7)	χ² = 0.07, p = 0.792	χ² = 3.24, p = 0.072
		Allele A, n (%)	49 (33.1)	52 (31.7)	21 (22.3)		
BIN1	rs10200967	CC, n (%)	25 (35.7)	33 (42.3)	29 (63.0)	χ² = 1.389, p = 0.499	**χ² = 12.61, p = 0.002**
		CT, n (%)	43 (61.4)	41 (52.6)	13 (28.3)		
		TT, n (%)	2 (2.9)	4 (5.1)	4 (8.7)		
		HWE χ² (p)	**9.96 (0.007)**	3.77 (0.151)	1.80 (0.407)		
		Call rate (%)	87.5	90.7	95.8		
		Allele C, n (%)	93 (66.4)	107 (68.6)	71 (77.2)	χ² = 0.16, p = 0.692	χ² = 3.09, p = 0.079
		Allele T, n (%)	47 (33.6)	49 (31.4)	21 (22.8)		
VDR	rs1544410	CC, n (%)	37 (46.8)	37 (45.1)	20 (44.4)	χ² = 2.10, p = 0.349	χ² = 3.20, p = 0.202
		CT, n (%)	29 (36.7)	37 (45.1)	22 (48.9)		
		TT, n (%)	13 (16.5)	8 (9.8)	3 (6.7)		

Gene	SNP	Genotype/Allele	Control (n=80)	MCI (n=86)	Dementia (n=48)	MCI vs Control χ^2 (p)	Dem vs Control χ^2 (p)
		HWE χ^2 (p)	2.89 (0.236)	0.08 (0.961)	0.89 (0.640)		
		Call rate (%)	98.8	95.3	93.8		
		Allele C, n (%)	103 (65.2)	111 (67.7)	62 (68.9)	χ^2 = 0.22, p = 0.636	χ^2 = 0.35, p = 0.553
		Allele T, n (%)	55 (34.8)	53 (32.3)	28 (31.1)		
VDR	rs7975232	AA, n (%)	22 (29.7)	20 (24.7)	10 (21.7)	χ^2 = 0.69, p = 0.707	χ^2 = 2.71, p = 0.258
		AC, n (%)	32 (43.2)	40 (49.4)	27 (58.7)		
		CC, n (%)	20 (27.0)	21 (25.9)	9 (19.6)		
		HWE χ^2 (p)	1.34 (0.512)	0.01 (0.994)	1.40 (0.496)		
		Call rate (%)	92.5	94.2	95.8		
		Allele A, n (%)	76 (51.4)	80 (49.4)	47 (51.1)	χ^2 = 0.12, p = 0.729	χ^2 = 0.00, p = 0.968
		Allele C, n (%)	72 (48.6)	82 (50.6)	45 (48.9)		
CLU	rs9331888	CC, n (%)	26 (35.6)	35 (42.2)	20 (41.7)	χ^2 = 0.97, p = 0.614	χ^2 = 2.71, p = 0.258
		CG, n (%)	33 (45.2)	36 (43.4)	24 (50.0)		
		GG, n (%)	14 (19.2)	12 (14.5)	4 (8.3)		
		HWE χ^2 (p)	0.37 (0.833)	0.30 (0.859)	0.75 (0.688)		

Gene	SNP	Genotype/Allele	Control (n=80)	MCI (n=86)	Dementia (n=48)	MCI vs Control χ^2 (p)	Dem vs Control χ^2 (p)
		Call rate (%)	91.3	96.5	100.0		
		Allele C, n (%)	85 (58.2)	106 (63.9)	64 (66.7)	χ^2 = 1.04, p = 0.308	χ^2 = 1.75, p = 0.186
		Allele G, n (%)	61 (41.8)	60 (36.1)	32 (33.3)		
CLU	rs11136000	CC, n (%)	30 (47.6)	28 (40.0)	15 (34.1)	χ^2 = 3.57, p = 0.168	χ^2 = 2.29, p = 0.319
		CT, n (%)	27 (42.9)	27 (38.6)	22 (50.0)		
		TT, n (%)	6 (9.5)	15 (21.4)	7 (15.9)		
		HWE χ^2 (p)	0.00 (0.999)	2.83 (0.243)	0.05 (0.975)		
		Call rate (%)	78.8	81.4	91.7		
		Allele C, n (%)	87 (69.0)	83 (59.3)	52 (59.1)	χ^2 = 2.74, p = 0.098	χ^2 = 2.26, p = 0.133
		Allele T, n (%)	39 (31.0)	57 (40.7)	36 (40.9)		
CR1	rs6656401	GG, n (%)	41 (59.4)	46 (59.7)	24 (53.3)	χ^2 = 0.27, p = 0.872	χ^2 = 0.83, p = 0.660
		GA, n (%)	22 (31.9)	26 (33.8)	18 (40.0)		
		AA, n (%)	6 (8.7)	5 (6.5)	3 (6.7)		
		HWE χ^2 (p)	1.38 (0.502)	0.25 (0.881)	0.02 (0.988)		
		Call rate (%)	86.3	89.5	93.8		

Gene	SNP	Genotype/Allele	Control (n=80)	MCI (n=86)	Dementia (n=48)	MCI vs Control χ^2 (p)	Dem vs Control χ^2 (p)
		Allele G, n (%)	104 (75.4)	118 (76.6)	66 (73.3)	$\chi^2 = 0.06$, p = 0.801	$\chi^2 = 0.12$, p = 0.731
		Allele A, n (%)	34 (24.6)	36 (23.4)	24 (26.7)		
CR1	rs1408077	CC, n (%)	46 (59.7)	53 (63.1)	21 (48.8)	$\chi^2 = 0.30$, p = 0.860	$\chi^2 = 1.64$, p = 0.439
		CA, n (%)	25 (32.5)	26 (31.0)	19 (44.2)		
		AA, n (%)	6 (7.8)	5 (6.0)	3 (7.0)		
		HWE χ^2 (p)	0.94 (0.623)	0.55 (0.761)	0.220 (0.896)		
		Call rate (%)	96.3	97.7	89.6		
		Allele C, n (%)	117 (76,0)	132 (78,6)	61 (70,9)	$\chi^2 = 0.31$, p = 0.578	$\chi^2 = 0.73$, p = 0.392
		Allele A, n (%)	37 (24,0)	36 (21,4)	25 (29,1)		
PICALM	rs541458	TT, n (%)	29 (44.6)	44 (56.4)	25 (55.6)	$\chi^2 = 2.17$, p = 0.338	$\chi^2 = 2.00$, p = 0.368
		TC, n (%)	27 (41.5)	27 (34.6)	17 (37.8)		
		CC, n (%)	9 (13.8)	7 (9.0)	3 (6.7)		
		HWE χ^2 (p)	0.44 (0.802)	0.89 (0.642)	0.00 (0.999)		
		Call rate (%)	81.3	90.7	93.8		
		Allele T, n (%)	85 (65.4)	115 (73.7)	67 (74.4)	$\chi^2 = 2.34$, p = 0.126	$\chi^2 = 2.04$, p = 0.153

Gene	SNP	Genotype/Allele	Control (n=80)	MCI (n=86)	Dementia (n=48)	MCI vs Control χ^2 (p)	Dem vs Control χ^2 (p)
		Allele C, n (%)	45 (34.6)	41 (26.3)	23 (25.6)		

Supplementary Table S3. Full results of association analysis for all 15 SNPs under four genetic models in females with MCI or dementia compared with controls.

Gene	SNP	Comparison	Model	Genotype	OR (95% CI)	p	q (FDR)
TOMM40	rs2075650	MCI vs Control	Codominant	AG vs AA	0.862 (0.451–1.650)	0.655	0.915
				GG vs AA	1.157 (0.245–5.466)	0.854	0.922
			Dominant	AG+GG vs AA	0.890 (0.475–1.665)	0.715	0.915
			Recessive	GG vs AA+AG	1.223 (0.264–5.668)	0.797	0.915
			Overdominant	AG vs AA+GG	0.854 (0.450–1.621)	0.629	0.907
		Dementia vs Control	Codominant	AG vs AA	0.765 (0.324–1.804)	0.541	0.853
				GG vs AA	0.700 (0.069–7.129)	0.764	0.915
			Dominant	AG+GG vs AA	0.759 (0.330–1.748)	0.517	0.853
			Recessive	GG vs AA+AG	0.772 (0.077–7.703)	0.825	0.922
			Overdominant	AG vs AA+GG	0.779 (0.333–1.824)	0.565	0.856
SORL1	rs2070045	MCI vs Control	Codominant	TG vs TT	0.534 (0.266–1.073)	0.078	0.835
				GG vs TT	0.983 (0.311–3.105)	0.977	0.984
			Dominant	TG+GG vs TT	0.599 (0.309–1.162)	0.129	0.835
			Recessive	GG vs TT+TG	1.344 (0.450–4.014)	0.597	0.877
			Overdominant	TG vs TT+GG	0.536 (0.276–1.042)	0.066	0.835
		Dementia vs Control	Codominant	TG vs TT	0.744 (0.337–1.639)	0.463	0.847
				GG vs TT	0.621 (0.138–2.791)	0.534	0.853
			Dominant	TG+GG vs TT	0.726 (0.338–1.561)	0.412	0.835
			Recessive	GG vs TT+TG	0.729 (0.172–3.083)	0.667	0.915
			Overdominant	TG vs TT+GG	0.801 (0.376–1.707)	0.565	0.856
SORL1	rs3824968	MCI vs Control	Codominant	TA vs TT	0.666 (0.338–1.312)	0.240	0.835
				AA vs TT	1.453 (0.554–3.815)	0.448	0.847
			Dominant	TA+AA vs TT	0.803 (0.424–1.522)	0.502	0.853
			Recessive	AA vs TT+TA	1.824 (0.752–4.428)	0.184	0.835
			Overdominant	TA vs TT+AA	0.599 (0.322–1.116)	0.106	0.835
		Dementia vs Control	Codominant	TA vs TT	0.872 (0.375–2.026)	0.750	0.915
				AA vs TT	1.484 (0.455–4.839)	0.513	0.853
			Dominant	TA+AA vs TT	0.980 (0.440–2.182)	0.960	0.979
			Recessive	AA vs TT+TA	1.609 (0.550–4.707)	0.385	0.835

Gene	SNP	Comparison	Model	Genotype	OR (95% CI)	p	q (FDR)
			Overdominant	TA vs TT+AA	0.778 (0.362–1.670)	0.520	0.853
SORL1	rs2282649	MCI vs Control	Codominant	CT vs CC	0.621 (0.306–1.261)	0.187	0.835
				TT vs CC	1.676 (0.548–5.122)	0.365	0.835
			Dominant	CT+TT vs CC	0.752 (0.382–1.478)	0.408	0.835
			Recessive	TT vs CC+CT	2.212 (0.784–6.239)	0.134	0.835
			Overdominant	CT vs CC+TT	0.547 (0.284–1.054)	0.071	0.835
		Dementia vs Control	Codominant	CT vs CC	0.907 (0.405–2.032)	0.813	0.918
				TT vs CC	1.209 (0.313–4.663)	0.783	0.915
			Dominant	CT+TT vs CC	0.946 (0.433–2.071)	0.890	0.940
			Recessive	TT vs CC+CT	1.284 (0.367–4.496)	0.696	0.915
			Overdominant	CT vs CC+TT	0.870 (0.412–1.839)	0.716	0.915
SORL1	rs1010159	MCI vs Control	Codominant	TC vs TT	0.658 (0.308–1.407)	0.280	0.835
				CC vs TT	1.657 (0.582–4.720)	0.344	0.835
			Dominant	TC+CC vs TT	0.811 (0.391–1.683)	0.574	0.861
			Recessive	CC vs TT+TC	2.196 (0.882–5.465)	0.091	0.835
			Overdominant	TC vs TT+CC	0.545 (0.282–1.055)	0.072	0.835
		Dementia vs Control	Codominant	TC vs TT	0.760 (0.317–1.823)	0.539	0.853
				CC vs TT	1.307 (0.386–4.424)	0.667	0.915
			Dominant	TC+CC vs TT	0.846 (0.364–1.969)	0.698	0.915
			Recessive	CC vs TT+TC	1.578 (0.545–4.570)	0.401	0.835
			Overdominant	TC vs TT+CC	0.693 (0.323–1.484)	0.345	0.835
BIN1	rs744373	MCI vs Control	Codominant	AG vs AA	0.889 (0.426–1.854)	0.754	0.915
				GG vs AA	3.129 (0.603–16.225)	0.174	0.835
			Dominant	AG+GG vs AA	1.034 (0.507–2.108)	0.927	0.959
			Recessive	GG vs AA+AG	3.327 (0.672–16.476)	0.141	0.835
			Overdominant	AG vs AA+GG	0.766 (0.377-1.556)	0.461	0.847
		Dementia vs Control	Codominant	AG vs AA	0.530 (0.233–1.206)	0.130	0.835
				GG vs AA	1.904 (0.317–11.423)	0.481	0.853
			Dominant	AG+GG vs AA	0.618 (0.282–1.357)	0.231	0.835
			Recessive	GG vs AA+AG	2.546 (0.441–14.686)	0.296	0.835
			Overdominant	AG vs AA+GG	0.498 (0.223–1.110)	0.088	0.835
BIN1	rs7561528	MCI vs Control	Codominant	GA vs GG	0.778 (0.403–1.500)	0.453	0.847

Gene	SNP	Comparison	Model	Genotype	OR (95% CI)	p	q (FDR)
				AA vs GG	1.348 (0.294–6.178)	0.701	0.915
			Dominant	GA+AA vs GG	0.815 (0.428–1.553)	0.534	0.853
			Recessive	AA vs GG+GA	1.557 (0.357–6.804)	0.556	0.856
			Overdominant	GA vs GG+AA	0.752 (0.398–1.421)	0.381	0.835
		Dementia vs Control	Codominant	GA vs GG	**0.282 (0.126–0.632)**	**0.002**	0.075
				AA vs GG	1.248 (0.256–6.080)	0.784	0.915
			Dominant	GA+AA vs GG	**0.345 (0.162–0.736)**	**0.006**	0.150
			Recessive	AA vs GG+GA	2.207 (0.471–10.343)	0.315	0.835
			Overdominant	GA vs GG+AA	**0.275 (0.125–0.606)**	**0.001**	0.050
BIN1	rs10200967	MCI vs Control	Codominant	CT vs CC	0.714 (0.363–1.404)	0.329	0.835
				TT vs CC	1.516 (0.255–9.025)	0.647	0.915
			Dominant	CT+TT vs CC	0.750 (0.385–1.461)	0.398	0.835
			Recessive	TT vs CC+CT	1.847 (0.325–10.498)	0.489	0.853
			Overdominant	CT vs CC+TT	0.688 (0.356–1.329)	0.266	0.835
		Dementia vs Control	Codominant	CT vs CC	**0.260 (0.114–0.590)**	**0.001**	0.050
				TT vs CC	1.731 (0.292–10.271)	0.546	0.853
			Dominant	CT+TT vs CC	**0.325 (0.150–0.704)**	**0.004**	0.120
			Recessive	TT vs CC+CT	3.252 (0.570–18.550)	0.184	0.835
			Overdominant	CT vs CC+TT	**0.246 (0.110–0.550)**	**0.001**	0.050
VDR	rs1544410	MCI vs Control	Codominant	CT vs CC	1.252 (0.641–2.443)	0.511	0.853
				TT vs CC	0.618 (0.229–1.670)	0.343	0.835
			Dominant	CT+TT vs CC	1.055 (0.566–1.965)	0.866	0.928
			Recessive	TT vs CC+CT	0.557 (0.217–1.431)	0.224	0.835
			Overdominant	CT vs CC+TT	1.389 (0.737–2.618)	0.310	0.835
		Dementia vs Control	Codominant	CT vs CC	1.415 (0.649–3.085)	0.383	0.835
				TT vs CC	0.426 (0.108–1.675)	0.222	0.835
			Dominant	CT+TT vs CC	1.105 (0.529–2.309)	0.791	0.915
			Recessive	TT vs CC+CT	0.361 (0.097–1.345)	0.129	0.835
			Overdominant	CT vs CC+TT	1.663 (0.789–3.504)	0.181	0.835
VDR	rs7975232	MCI vs Control	Codominant	AC vs AA	1.406 (0.653–3.026)	0.384	0.835
				CC vs AA	1.153 (0.486–2.735)	0.746	0.915
			Dominant	AC+CC vs AA	1.308 (0.642–2.666)	0.459	0.847

Gene	SNP	Comparison	Model	Genotype	OR (95% CI)	p	q (FDR)
			Recessive	CC vs AA+AC	0.931 (0.454–1.906)	0.844	0.922
			Overdominant	AC vs AA+CC	1.310 (0.693–2.477)	0.405	0.835
		Dementia vs Control	Codominant	AC vs AA	1.860 (0.750–4.611)	0.180	0.835
				CC vs AA	0.990 (0.335–2.931)	0.986	0.986
			Dominant	AC+CC vs AA	1.523 (0.644–3.602)	0.338	0.835
			Recessive	CC vs AA+AC	0.657 (0.269–1.602)	0.355	0.835
			Overdominant	AC vs AA+CC	1.869 (0.885–3.947)	0.101	0.835
CLU	rs9331888	MCI vs Control	Codominant	CG vs CC	0.831 (0.414–1.668)	0.602	0.877
				GG vs CC	0.594 (0.234–1.511)	0.274	0.835
			Dominant	CG+GG vs CC	0.760 (0.397–1.455)	0.407	0.835
			Recessive	GG vs CC+CG	0.655 (0.277–1.545)	0.333	0.835
			Overdominant	CG vs CC+GG	0.966 (0.510–1.829)	0.915	0.953
		Dementia vs Control	Codominant	CG vs CC	0.957 (0.436–2.104)	0.914	0.953
				GG vs CC	0.360 (0.102–1.272)	0.113	0.835
			Dominant	CG+GG vs CC	0.776 (0.367–1.638)	0.505	0.853
			Recessive	GG vs CC+CG	0.369 (0.113–1.210)	0.100	0.835
			Overdominant	CG vs CC+GG	1.229 (0.590–2.561)	0.582	0.864
CLU	rs11136000	MCI vs Control	Codominant	CT vs CC	1.102 (0.522–2.324)	0.799	0.915
				TT vs CC	2.756 (0.932–8.143)	0.067	0.835
			Dominant	CT+TT vs CC	1.402 (0.701–2.804)	0.339	0.835
			Recessive	TT vs CC+CT	2.627 (0.947–7.292)	0.064	0.835
			Overdominant	CT vs CC+TT	0.853 (0.425–1.712)	0.654	0.915
		Dementia vs Control	Codominant	CT vs CC	1.650 (0.713–3.819)	0.243	0.835
				TT vs CC	2.362 (0.673–8.297)	0.180	0.835
			Dominant	CT+TT vs CC	1.779 (0.801–3.951)	0.157	0.835
			Recessive	TT vs CC+CT	1.807 (0.562–5.806)	0.320	0.835
			Overdominant	CT vs CC+TT	1.344 (0.619–2.916)	0.455	0.847
CR1	rs6656401	MCI vs Control	Codominant	GA vs GG	1.072 (0.527–2.179)	0.848	0.922
				AA vs GG	0.797 (0.224–2.841)	0.727	0.915
			Dominant	GA+AA vs GG	1.015 (0.521–1.975)	0.966	0.979
			Recessive	AA vs GG+GA	0.777 (0.224–2.696)	0.691	0.915
			Overdominant	GA vs GG+AA	1.100 (0.549–2.204)	0.788	0.915

Gene	SNP	Comparison	Model	Genotype	OR (95% CI)	p	q (FDR)
		Dementia vs Control	Codominant	GA vs GG	1.403 (0.629–3.131)	0.408	0.835
				AA vs GG	0.866 (0.196–3.818)	0.849	0.922
			Dominant	GA+AA vs GG	1.291 (0.603–2.762)	0.511	0.853
			Recessive	AA vs GG+GA	0.756 (0.178–3.216)	0.705	0.915
			Overdominant	GA vs GG+AA	1.428 (0.653–3.124)	0.372	0.835
CR1	rs1408077	MCI vs Control	Codominant	CA vs CC	0.933 (0.472–1.845)	0.843	0.922
				AA vs CC	0.821 (0.229–2.947)	0.762	0.915
			Dominant	CA+AA vs CC	0.913 (0.479–1.739)	0.782	0.915
			Recessive	AA vs CC+CA	0.840 (0.240–2.939)	0.785	0.915
			Overdominant	CA vs CC+AA	0.953 (0.489–1.857)	0.887	0.940
		Dementia vs Control	Codominant	CA vs CC	1.644 (0.743–3.638)	0.220	0.835
				AA vs CC	1.052 (0.232–4.760)	0.948	0.974
			Dominant	CA+AA vs CC	1.536 (0.716–3.293)	0.270	0.835
			Recessive	AA vs CC+CA	0.839 (0.194–3.629)	0.814	0.918
			Overdominant	CA vs CC+AA	1.633 (0.756–3.528)	0.212	0.835
PICALM	rs541458	MCI vs Control	Codominant	TC vs TT	0.676 (0.331–1.379)	0.281	0.835
				CC vs TT	0.519 (0.173–1.552)	0.240	0.835
			Dominant	TC+CC vs TT	0.636 (0.327–1.238)	0.183	0.835
			Recessive	CC vs TT+TC	0.614 (0.215–1.757)	0.363	0.835
			Overdominant	TC vs TT+CC	0.763 (0.386–1.509)	0.437	0.847
		Dementia vs Control	Codominant	TC vs TT	0.736 (0.327–1.657)	0.459	0.847
				CC vs TT	0.388 (0.094–1.592)	0.189	0.835
			Dominant	TC+CC vs TT	0.648 (0.301–1.396)	0.268	0.835
			Recessive	CC vs TT+TC	0.444 (0.113–1.743)	0.245	0.835
			Overdominant	TC vs TT+CC	0.861 (0.394–1.881)	0.708	0.915

Supplementary Table S4. Full results of association analysis for all 15 SNPs under four genetic models in females with dementia compared with MCI.

Gene	SNP	Comparison	Model	Genotype	OR (95% CI)	p	q (FDR)
TOMM40	rs2075650	Dementia vs MCI	Codominant	AG vs AA	0.873 (0.369–2.066)	0.758	0.931
				GG vs AA	0.654 (0.068–6.263)	0.713	0.931
			Dominant	AG+GG vs AA	0.848 (0.369–1.951)	0.699	0.931
			Recessive	GG vs AA+AG	0.684 (0.073–6.437)	0.740	0.931
			Overdominant	AG vs AA+GG	0.893 (0.380–2.099)	0.795	0.931
SORL1	rs2070045	Dementia vs MCI	Codominant	TG vs TT	1.399 (0.647–3.027)	0.394	0.931
				GG vs TT	0.614 (0.148–2.546)	0.501	0.931
			Dominant	TG+GG vs TT	1.212 (0.581–2.531)	0.608	0.931
			Recessive	GG vs TT+TG	0.525 (0.133–2.079)	0.359	0.931
			Overdominant	TG vs TT+GG	1.507 (0.715–3.173)	0.281	0.931
SORL1	rs3824968	Dementia vs MCI	Codominant	TA vs TT	1.339 (0.576–3.109)	0.497	0.931
				AA vs TT	1.030 (0.345–3.081)	0.957	0.979
			Dominant	TA+AA vs TT	1.240 (0.565–2.725)	0.592	0.931
			Recessive	AA vs TT+TA	0.880 (0.327–2.371)	0.801	0.931
			Overdominant	TA vs TT+AA	1.326 (0.618–2.842)	0.469	0.931
SORL1	rs2282649	Dementia vs MCI	Codominant	CT vs CC	1.465 (0.663–3.238)	0.345	0.931
				TT vs CC	0.719 (0.215–2.398)	0.591	0.931
			Dominant	CT+TT vs CC	1.258 (0.590–2.682)	0.552	0.931
			Recessive	TT vs CC+CT	0.578 (0.190–1.758)	0.334	0.931
			Overdominant	CT vs CC+TT	1.599 (0.769–3.324)	0.209	0.931
SORL1	rs1010159	Dementia vs MCI	Codominant	TC vs TT	1.161 (0.498–2.706)	0.729	0.931
				CC vs TT	0.775 (0.262–2.295)	0.646	0.931
			Dominant	TC+CC vs TT	1.039 (0.465–2.324)	0.925	0.979
			Recessive	CC vs TT+TC	0.704 (0.276–1.799)	0.464	0.931
			Overdominant	TC vs TT+CC	1.286 (0.620–2.671)	0.499	0.931
BIN1	rs744373	Dementia vs MCI	Codominant	AG vs AA	0.606 (0.271–1.352)	0.221	0.931
				GG vs AA	0.611 (0.162–2.299)	0.466	0.931
			Dominant	AG+GG vs AA	1.295 (0.607–0.284)	0.196	0.931
			Recessive	GG vs AA+AG	0.768 (0.215–2.751)	0.686	0.931

Gene	SNP	Comparison	Model	Genotype	OR (95% CI)	p	q (FDR)
			Overdominant	AG vs AA+GG	0.659 (0.305–1.427)	0.290	0.931
BIN1	rs7561528	Dementia vs MCI	Codominant	GA vs GG	**0.371 (0.168–0.820)**	**0.014**	0.281
				AA vs GG	0.959 (0.234–3.927)	0.954	0.979
			Dominant	GA+AA vs GG	**0.433 (0.206–0.910)**	**0.027**	0.400
			Recessive	AA vs GG+GA	1.461 (0.369–5.785)	0.589	0.931
			Overdominant	GA vs GG+AA	**0.372 (0.172–0.809)**	**0.013**	0.281
BIN1	rs10200967	Dementia vs MCI	Codominant	CT vs CC	**0.369 (0.165–0.823)**	**0.015**	0.281
				TT vs CC	1.180 (0.268–5.194)	0.826	0.939
			Dominant	CT+TT vs CC	**0.440 (0.207–0.934)**	**0.032**	0.400
			Recessive	TT vs CC+CT	1.827 (0.430–7.765)	0.414	0.931
			Overdominant	CT vs CC+TT	**0.362 (0.165–0.792)**	**0.011**	0.281
VDR	rs1544410	Dementia vs MCI	Codominant	CT vs CC	1.105 (0.516–2.364)	0.797	0.931
				TT vs CC	0.659 (0.156–2.790)	0.571	0.931
			Dominant	CT+TT vs CC	1.024 (0.491–2.132)	0.950	0.979
			Recessive	TT vs CC+CT	0.626 (0.156–2.512)	0.509	0.931
			Overdominant	CT vs CC+TT	1.178 (0.567–2.448)	0.661	0.931
VDR	rs7975232	Dementia vs MCI	Codominant	AC vs AA	1.336 (0.541–3.301)	0.530	0.931
				CC vs AA	0.866 (0.291–2.582)	0.797	0.931
			Dominant	AC+CC vs AA	1.176 (0.495–2.794)	0.714	0.931
			Recessive	CC vs AA+AC	0.708 (0.292–1.716)	0.444	0.931
			Overdominant	AC vs AA+CC	1.433 (0.688–2.985)	0.336	0.931
CLU	rs9331888	Dementia vs MCI	Codominant	CG vs CC	1.145 (0.537–2.442)	0.725	0.931
				GG vs CC	0.587 (0.167–2.067)	0.407	0.931
			Dominant	CG+GG vs CC	1.006 (0.488–2.073)	0.988	0.988
			Recessive	GG vs CC+CG	0.547 (0.166–1.804)	0.321	0.931
			Overdominant	CG vs CC+GG	1.280 (0.625–2.620)	0.500	0.931
CLU	rs11136000	Dementia vs MCI	Codominant	CT vs CC	1.507 (0.648–3.506)	0.341	0.931
				TT vs CC	0.854 (0.285–2.561)	0.778	0.931
			Dominant	CT+TT vs CC	1.273 (0.579–2.800)	0.548	0.931
			Recessive	TT vs CC+CT	0.683 (0.253–1.841)	0.451	0.931
			Overdominant	CT vs CC+TT	1.589 (0.740–3.408)	0.235	0.931
CR1	rs6656401	Dementia vs MCI	Codominant	GA vs GG	1.295 (0.592–2.831)	0.518	0.931

Gene	SNP	Comparison	Model	Genotype	OR (95% CI)	p	q (FDR)
				AA vs GG	1.143 (0.250–5.217)	0.863	0.966
			Dominant	GA+AA vs GG	1.270 (0.602–2.678)	0.530	0.931
			Recessive	AA vs GG+GA	1.033 (0.234–4.562)	0.966	0.979
			Overdominant	GA vs GG+AA	1.277 (0.594–2.744)	0.532	0.931
CR1	rs1408077	Dementia vs MCI	Codominant	CA vs CC	1.722 (0.780–3.800)	0.179	0.931
				AA vs CC	1.347 (0.287–6.312)	0.706	0.931
			Dominant	CA+AA vs CC	1.662 (0.777–3.556)	0.190	0.931
			Recessive	AA vs CC+CA	1.060 (0.235–4.786)	0.939	0.979
			Overdominant	CA vs CC+AA	1.664 (0.770–3.592)	0.195	0.931
PICALM	rs541458	Dementia vs MCI	Codominant	TC vs TT	1.102 (0.504–2.410)	0.807	0.931
				CC vs TT	0.750 (0.178–3.169)	0.696	0.931
			Dominant	TC+CC vs TT	1.030 (0.491–2.159)	0.938	0.979
			Recessive	CC vs TT+TC	0.722 (0.177–2.949)	0.650	0.931
			Overdominant	TC vs TT+CC	1.142 (0.532–2.450)	0.734	0.931
